# Role of Epstein–Barr Virus in the Pathogenesis of Head and Neck Cancers and Its Potential as an Immunotherapeutic Target

**DOI:** 10.3389/fonc.2018.00257

**Published:** 2018-07-06

**Authors:** Queenie Fernandes, Maysaloun Merhi, Afsheen Raza, Varghese Philipose Inchakalody, Nassima Abdelouahab, Abdul Rehman Zar Gul, Shahab Uddin, Said Dermime

**Affiliations:** ^1^Translational Cancer Research Facility, Hamad Medical Corporation, Doha, Qatar; ^2^National Center for Cancer Care and Research, Hamad Medical Corporation, Doha, Qatar; ^3^Interim Translational Research Institute, Hamad Medical Corporation, Doha, Qatar

**Keywords:** Epstein–Barr virus, head and neck cancers, nasopharyngeal cancer, EBV-induced nuclear antigen 1, LMP, cancer vaccine, virus-specific T cells, cancer immunotherapy

## Abstract

The role of Epstein–Barr virus (EBV) infection in the development and progression of tumor cells has been described in various cancers. Etiologically, EBV is a causative agent in certain variants of head and neck cancers such as nasopharyngeal cancer. Proteins expressed by the EVB genome are involved in invoking and perpetuating the oncogenic properties of the virus. However, these protein products were also identified as important targets for therapeutic research in the past decades, particularly within the context of immunotherapy. The adoptive transfer of EBV-targeted T-cells as well as the development of EBV vaccines has opened newer lines of research to conceptualize novel therapeutic approaches toward the disease. This review addresses the most important aspects of the association of EBV with head and neck cancers from an immunological perspective. It also aims to highlight the current and future prospects of enhanced EBV-targeted immunotherapies.

## Introduction

Head and neck cancers represent a distinct group of cancers occurring in the pharyngeal, laryngeal, nasopharyngeal, and oropharyngeal regions, the salivary glands, as well as the oral and nasal cavities. Head and neck cancer is one of the most frequently observed tumors in the world (1). The incidence and distribution of each tumor type is often dependent on the geographical location, population diversity, and level of exposure to the risk factors. Tobacco smoking and consumption of alcohol are identified as the major risk factors leading to the disease. It is reported that out of the 72% of head and neck cancers caused by tobacco and alcohol consumption, 33% of the cases were caused by tobacco alone, 4% cases were caused due to drinking alcohol, and the remaining 35% cases were caused by the combined indulgence in both (2). Although this cancer is classically known to be tobacco and alcohol induced, most cases can be caused by infection through certain viruses like the human papilloma virus or the Epstein–Barr virus (EBV) (1).

Epstein–Barr virus is known to belong to a family of the herpes virus. It was identified as early as 1964 by Epstein’s group in a Burkett’s lymphoma cell line, and hence its nomenclature. The presence of the virus is ubiquitous as nearly 90% of the human adult population is said to be infected by the virus (3, 4). Transmission of the virus causing head and neck cancers is known to mainly occur through saliva (5).

This review intends to shed light on the role of EBV in the pathogenesis of the head and neck carcinomas and the most important immunological aspects underlying the infection. It also highlights the use of immunotherapeutic interventions as a potential modality for targeting EBV-associated head and neck cancers.

## EBV-Induced Oncogenic Infection

Many viral infections are known to occur during early childhood. Most of these infections are often mild. However, infections that strike during adulthood can lead to infectious mononucleosis (3). It is a disease that is characterized by a triad of symptoms: pharyngitis, lymphadenopathy, and fever (4). Once an infection occurs, the individual becomes a lifelong carrier of the virus, often without any known symptoms to the disease.

The virus is capable of exhibiting dual tropism. This means that it can infect both, B cells and epithelial cells (6). Under latent conditions, the virus survives in the pool of infected memory B cells (7). Human B cells are more easily infected by the virus than the epithelial cells (8). The virus is capable of alternating its cell entry mechanisms to infect epithelial or B cells by switching its envelop proteins (8). EBV is known to engage the envelope protein gp350 to bind to the complement receptor type 2 protein which is found on the membrane surface of B cells. On the other hand, in epithelial cells, it switches to using the gp40 envelop protein to bind to the surface integrins (8). This shuttle used in different infection and cell entry mechanisms is critical to the EBV’s persistence in humans.

Plasma EBV deoxyribonucleic acid (DNA) is present in the tumor cells of almost all anaplastic nasopharyngeal cancers (NPCs) (9), and it is considered as the most accurate molecular predictive biomarker of disease diagnosis and response to treatment (10). Clinically, EBV-associated undifferentiated NPC is highly invasive and metastatic (11). Precision radiotherapy is used for the treatment of early stage NPC. However, conventional treatment in advanced stages includes chemo-radiotherapy with or without adjunct chemotherapy (12, 13).

## Oncogenic Pathogenesis

Epstein–Barr virus was identified as the first human virus to be linked to carcinogenesis (14). Since then it was classified as a group 1 carcinogen (5, 15). It is commonly known to immortalize normal B cells *in vitro*. EBV can mediate infection *via* two mechanisms. Usually, the virus remains latent without inflicting any symptoms. However, sometimes, the virus can revert to a lytic state causing the transformation of cells into malignant tumors (16). Moreover, its viral gene products are known to be expressed in almost all EBV-associated cancers at a molecular level. The expressed viral proteins are known to trigger oncogenesis by blocking apoptosis, facilitating genomic instabilities, and inducing uncontrolled cell proliferation and migration. These events are precisely known to mark tumor initiation followed by sustained tumor maintenance (17). Upon oncogenic transformation of cells, EBV is known to display typical mechanisms to escape immune recognition, thereby promoting oncogenesis and tumor progression. For example, EBV is known to express very few of its genes upon the initial lytic infection to prevent detection by the host’s immune system (18). The virus is also known to exert a number of other immunomodulatory effects like the silencing of the anti-EBV effect of interferon-gamma (INF-γ) in B cells. In addition, it mediates changes in the production of certain antiviral cytokines like TNF-α, IL-1β, and IL-6 (19). Another EBV cytokine that is able to mimic the characteristics of IL-10 permits the virus to escape the host’s antiviral response (19, 20). Synergistically, a compromised host–immune system owing to certain other medical conditions and a chronic inflammatory host–microenvironment are also known to enhance the malignant pathogenesis of the virus (21).

## EBV Protein Expression

**Figure 1:**
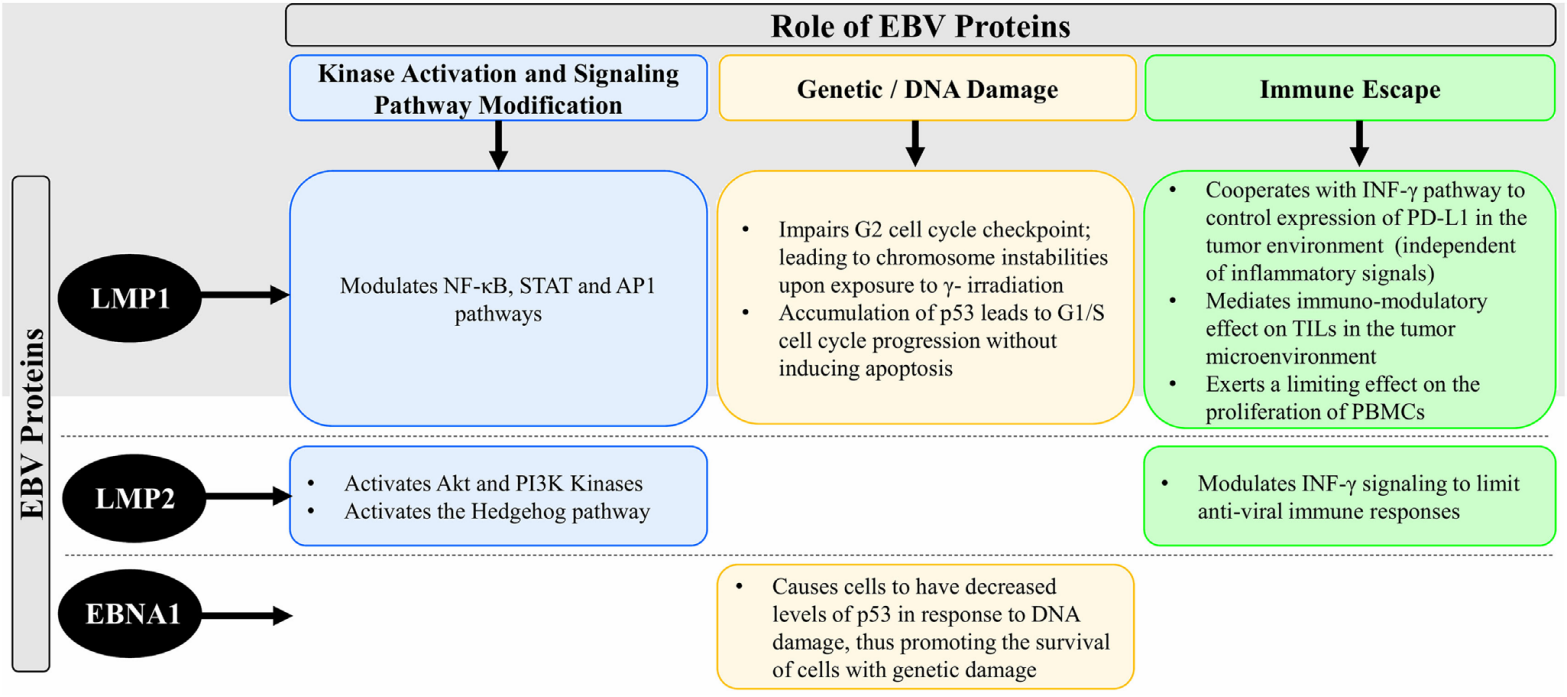
Schematic diagram comparing the role of the EBV proteins (LMP1, LMP2, and EBNA1) in the oncogenic pathogenesis and/or the immune escape of NPC. Abbreviations: EBV, Epstein–Barr virus; LMP1, latent membrane protein 1; LMP2, latent membrane protein 2; EBNA1, EBV-induced nuclear antigen 1; NF-κB, nuclear factor kappa-light-chain-enhancer of activated B cells; STAT, signal transducer and activator of transcription; AP1, activator protein 1; Akt, protein kinase B; PI3K, phosphatidylinositol-4,5-bisphosphate 3-kinase; p53, cellular tumor antigen p53; INF-γ, interferon-gamma; TILs, tumor-infiltrating lymphocytes; PD-L1, programmed cell death protein 1 ligand; PBMCs, peripheral blood mononuclear cells; DNA, deoxyribonucleic acid; NPC, nasopharyngeal cancer.

Epstein–Barr virus that is particularly present in NPC is restricted to the expression of viral latent genes to produce the EBV-induced nuclear antigen 1 (EBNA1) protein and the latent membrane proteins [latent membrane protein 1 (LMP1), LMP2A, and LMP2B] in addition to other EBV-encoded small RNAs and Bam H1 A rightward transcript (BART) microRNAs (miRNAs). Table 1 summarizes the EBV-associated/linked proteins and miRNAs involved in head and neck cancers pathogenesis. Each of these proteins is translated from the viral genome to serve a particular and a distinct purpose in inflicting oncogenic transformation in cancers of the head and neck regions. Figure 1 compares the role of the three EBV proteins (LMP1, LMP2, and EBNA1) in the oncogenic pathogenesis and/or the immune escape of NPCs.

**Table 1:** EBV-associated proteins and miRNAs involved in the pathogenesis of NPC.

EBV proteins	Additional/supporting roles in promoting the oncogenic pathogenesis of NPC
LMP1	Promotes expression of anti-apoptotic proteins (22) Stimulates cell growth by upregulating cell growth factor receptors (23) Induces an epithelial to mesenchymal transition in cancer cells (24, 25) Secretes MMPs that facilitate the degradation of the extracellular matrix, thereby making cells susceptible to the virus (26–29) Modulates the stability of p53; a major regulator of tumor progression (30) Regulates the reactive binding of nuclear expressed EGFR to cell cycle promoters (31) Overexpression is found to regulate angiogenesis, thereby causing NPC tumors to display a higher concentration of microvessels (32)

LMP2	Promotes cancer cell migration and invasion (33, 34) Counteracts pro-apoptotic effects of TGF-β1 through PI3K–Akt pathway (35) Linked to anchorage-independent growth observed in soft agar (35, 36) Potentiates cancer stem cell like properties through the activation of the hedgehog signaling pathway (22)

EBNA1	Maintains the stability of the EBV genomes in the infected cells (37) Reduces p53 levels and promotes cell survival (38) Suppresses TGF-β1 signaling and promotes oncogenesis (39) Expressed in memory B cells undergoing division (40) Inactivation reduces the copy number of the episomes in EBV-infected B lymphoma cells *in vitro* and inhibits growth (41) Overexpression increases the nuclear levels of metastatic proteins like mapsin, Nm23-H1, and stathmin1 in NPC (42)

BARTs	Increased expression of functional proteins in oncogenesis (43, 44) Varying expression levels indicate whether EBV infection is lytic or latent (45)

EBV-encoded miRNAs	miR-BART3-5p targets DICE1 which is a tumor suppressor gene in NPC (46) miR-BART9 promotes invasion and metastatic properties of NPC cells *in vitro* (47) miR-BART17-5p, miR-BART17-16, or miR-BART17-1-5p are known to target LMP1 (48) miR-BART22 is found to target LMP2 (49)

*EBV, Epstein–Barr virus; p53, cellular tumor antigen p53; EGFR, epidermal growth factor receptor; NPC, nasopharyngeal cancer; TGF-β1, transforming growth factor beta 1; PI3K, phosphatidylinositol-4,5-bisphosphate 3-kinase; Akt, protein kinase B; DICE1, deleted in cancer; LMP1, latent membrane protein 1; LMP2, latent membrane protein 2; EBNA1, EBV-induced nuclear antigen; BARTs, Bam H1 A rightward transcripts; miRNAs, microRNAs; MMPs, matrix metalloproteases*.

### Latent Membrane Protein 1

Latent membrane protein 1 is a 66-kDa integral transmembrane protein that is known to play an important role in promoting malignant transformation in NPC (37, 50). It has three distinct functional domains within its C-terminal region, namely, C-terminal activating regions 1, 2, and 3 (CTAR1, CTAR2, and CTAR3). Each of these functional domains regulates different signaling pathways in the pathogenesis of NPC (30). Within the context of NPC, LMP1 participates in the NF-κB, signal transducer and activator of transcription 3, and activator protein 1 signaling pathways (51, 52). Most LMP-mediated signal transduction events are mediated *via* the CTAR1 and CTAR2 functional domains, while the role of CTAR3 is still partially unknown. The combined activation of these pathways leads to the upregulation of the programmed cell death protein 1 ligand (PD-L1) (53) which is an important immune-checkpoint inhibitor in cancer immunology. This could also mean that different expression levels of LMP1 may trigger different signaling pathways. Interestingly, LMP1 is a viral mimic of CD40, a member of the TNFR family. This viral protein functions by inducing the expression of multiple cellular genes that play a role in regulating cell growth and apoptosis. It is also known to upregulate the expression of cancer stem cell markers leading to high metastatic features in NPCs (1). Cells that express LMP1 also exhibit an impaired G2 cell cycle checkpoint. This in turn leads to chromosome instabilities and chromatid breaks upon exposure to gamma-irradiation (54). NPC is known to be a highly metastatic cancer (55) in which LMP1 is able to enhance the invasion and migration potential of the cancer cells. It is also found to bring about an epithelial-to-mesenchymal transition in these cells (24, 25). LMP1 is known to facilitate cell invasion and tumorigenesis through the secretion of matrix metalloproteases (MMPs). These MMPs facilitate the degradation of the extracellular matrix, thereby making the cells susceptible to the virus (26–29).

The protein cellular tumor antigen p53 (p53) is a known tumor suppressor that mediates apoptosis. LMP1 is believed to modulate the stability of p53 thus highlighting its role in regulating tumor progression (30). In relation to this, a study was able to prove that LMP1 exposure of NPC cells led to the accumulation of p53 which in turn promoted G1/S cell cycle progression without inducing apoptosis (56). Another protein playing an important role in carcinogenesis is the epidermal growth factor receptor (EGFR). EGFR is often found to be localized to the nucleus in NPC cells (57–61). The reactive binding of this nuclear expressed EGFR to cell cycle promoters is also known to be regulated by LMP1 (31).

Another critical process regulated by LMP1 is angiogenesis. NPC tumors were shown to display a higher concentration of microvessels that was brought about by an overexpression of LMP1 (32).

Apart from its active contribution toward establishing and promoting oncogenesis and tumor progression, LMP1 is also known to passively promoter oncogenic transformation of cells through mediated immune escape (62–64). For example, LMP1 cooperates with INF-γ pathways to regulate the expression of PD-L1 independently of inflammatory signals in the tumor environment (53). EBV-positive tumors are known to actively secrete LMP1, which it mediates immunosuppressive effects on tumor-infiltrating lymphocytes in the tumor microenvironment. Another immunomodulatory role was identified by the ability of LMP1 containing exosomes to inhibit proliferation of peripheral blood mononuclear cells (PBMCs) (65). It is therefore evident that LMP1 plays a pivotal role in the immune regulation of NPC, hence mediating immunological escape of the cancer. On the other hand, it was demonstrated that low levels of LMP1 are associated with cell growth and survival, while high expression levels are noted to exhibit growth inhibition and sensitization to apoptosis in response to a varying stimulus (66, 67). However, the sole expression of the LMP1 gene in immortalized nasopharyngeal epithelial cells did not induce malignant transformation *in vitro* (50, 68, 69). These contradicting results may be due to the ability of LMP1 to upregulate both pro- and anti-apoptotic genes and disrupt DNA repair mechanisms (70–72).

### Latent Membrane Protein 2

Latent membrane protein 2 is another latent membrane protein expressed by the EBV genome. This group includes two proteins, namely, LMP2A and LMP2B. While these proteins may not be essential for the malignant transformation of B cells, LMP2A expression is critical for tumorigenesis of epithelial cells *in vitro* (73). LMP2 was found to be linked to anchorage-independent growth observed in soft agar (35, 36). The same study was also able to show that LMP2 could inhibit differentiation through the activation of the protein kinase B and PI2 kinases. Moreover, it is capable of potentiating cancer stem cell like properties *via* the activation of the hedgehog signaling pathway (22). Furthermore, LMP2 can modulate INF-γ signaling to limit antiviral immune responses against EBV, thereby mediating immune escape in cancer (74).

### EBV-Induced Nuclear Antigen 1

EBV-induced nuclear antigen 1 is solely expressed in memory B cells undergoing division (40). EBV-induced B cell lymphoma is a characteristic of type 1 latency, while type 2 latency is a characteristic of NPC. As EBNA1 is required for the preservation and persistence of the viral genome in latent infections, it is found to be expressed in all EBV-associated cancers including NPC (75). Its function is to help in the replication of the viral episomes, followed by their segregation into mitotic daughter cells. As demonstrated by a certain study (41), inactivating the function of EBNA1 is found to reduce the copy number of episomes in EBV-infected B lymphoma cells *in vitro*, which inhibits their growth. Another study targeting the profiling of the nuclear proteome of NPC cells reported that EBNA1 overexpression led to metastasis (42). This effect was mainly because mapsin, Nm23-H1, and stathmin1 are metastatic proteins whose nuclear levels were found to substantially increase upon the overexpression of EBNA1. In addition, another role of the EBNA1 protein was identified through its ability to promote the survival of cells with damaged DNA, thereby increasing the occurrence of chromosomal instabilities. This is not surprising because cells that express EBNA1 have decreased levels of p53 in response to DNA damage (8). Moreover, in NPC cells that express EBNA1, an increased expression of ROS and NAPDH oxidase levels were identified (42). This indicates that of the fact that EBNA1 advocates oxidative stress-induced DNA damage and further allowing the survival of these cells by destabilizing p53. EBNA1 is also capable of modulating a number of cellular pathways that target cell invasion, cell proliferation, survival, and DNA damage repair. In a particular study, expression of EBNA1 in HONE1 NPC cells was shown to trigger oncogenesis and promote metastasis in nude mice (76).

### Bam H1 A Rightward Transcripts

Bam H1 A rightward transcripts are RNA transcripts that are found rightwards from the BAMH1 A region of EBV genome (43, 77, 78). An abundance of BART expression is commonly observed in NPC (43, 44). This increased expression indicates that BARTs may encode for functional proteins in oncogenesis. However, there is still a lack of supporting evidence for the expression of endogenous BART proteins in EBV-infected cells (44, 79). It is also surprising to note that the expression levels of BART are known to vary depending on whether the infection is lytic or latent (45). These findings demand further detailed investigation to elucidate the potential roles of the BART proteins in the pathogenesis of EBV-induced NPC.

### EBV-Encoded miRNAs

Epstein–Barr virus is known to encode for around 44 miRNAs (80). miRNAs are short non-coding RNAs that act at the post-transcriptional level and are often linked to oncogenic pathogenesis (81). BART miRNA expression is a characteristic of EBV infection in almost all cell types. However, their expression levels are notably higher in epithelial cells as compared to B cells (82). Although complete knowledge is still not acquired on the possible targets of all BART miRNAs, a few key targets have been identified and their functions have been validated. The miR-BART3-5p is known to target deleted in cancer which is a tumor suppressor gene in NPC (46). Another study identified that miR-BART9 is capable of promoting invasion and metastatic properties of NPC cells *in vitro* (47). Moreover, it was interesting to notice that a few BART miRNAs can also directly target EBV viral proteins. For example, LMP1 is targeted by miR-BART17-5p, miR-BART17-16, or miR-BART17-1-5p (48), whereas LMP2 is targeted by miR-BART22 (49). Therefore, it is evident that EBV is able to direct oncogenic protein expression through the varying roles of BART miRNAs.

## Immunotherapeutic Interventions

### EBV Vaccines for NPC

The primary standard of care against EBV-associated NPC includes radiation and/or chemotherapy which serve as efficient therapeutic strategies (83). However, 15–30% of NPC patients show poor prognosis and develop failure at various sites, while 5–15% demonstrate local failure. Furthermore, side effects associated with radiotherapy and chemotherapy are common (12). Therefore, development of novel therapeutic agents with limited side effects and low off target toxicities are a focus of interest globally.

In NPC, a number of EBV-associated latent genes including non-coding RNA (EBER), EBV EBNA 1, LMP 1/LMP2, and BARTs are highly expressed by tumor cells. These EBV-associated proteins lead to latent EBV infection in NPC (84). From the perspective of immune responses, high protein expression and latent EBV infection should serve as an advantage in NPC as it should contribute to antitumor responses. Studies have shown that substantial immune infiltrates consisting of dendritic cells, monocytes, inflammatory cytokines, and T and B cells are observed in NPC tumors indicating the utility of these cells in tumor control (85, 86). By contrast, limited natural antitumor responses are observed in NPC leading to poor tumor control (87). It is postulated that immune-suppressive microenvironment and immune checkpoints/cytokines within the tumor site may contribute to functional inactivation of innate cytotoxic T cell responses. This was evidenced by the observation of heavy infiltration of lymphoid cells, predominantly CD4^+^CD25^high^ Foxp3^+^ regulatory T cells and myeloid-derived suppressor cells that may be involved in dampening naturally occurring immune responses and limiting antitumor responses (87, 88). Therefore, to counter the immune-suppressive microenvironment and to enhance EBV-specific immune responses, immunotherapeutic strategies are being explored in NPC.

Cancer immunotherapy in the form of vaccines has recently emerged as a promising and an effective modality to treat different malignancies. With respect to vaccine development against EBV-associated NPC, the goal seems attainable due to the distinct immune-biology of the virus and its association with the tumor cells (89). In EBV-associated NPC, EBV-specific proteins should serve as candidate targets for vaccine development and immune modulation (90, 91). To this end, the role of therapeutic vaccines has been tested in preclinical and clinical trials with promising results albeit some challenges (90). The main targets for vaccination strategies in NPC include the EBV-associated proteins LMP1, LMP2, and EBNA1 (91). Of these latent proteins, LMP2A and EBNA1 are considered the most promising targets for EBV-specific vaccine development due to their high expression levels (92). In NPC, EBNA1 is a critical protein as it maintains viral DNA in dividing cells and modulates cellular pathways. It exhibits various CD4^+^ T cell epitopes that makes this protein a distinct immunotherapeutic target (93, 94). Similarly, LMP2A is a transmembrane protein that possesses limited number of CD4^+^ epitopes but large number of CD8^+^ T-cell epitopes (95, 96). As such, LMP2A is considered as a prime CD8^+^ T cell target in NPC (89). Thus, both EBNA1 and LMP2 have been identified as attractive candidate vaccine targets in NPC due to their immunological competences as well as their ability to cause latent EBV infection (91). From an immunological perspective, latent EBV infection maintains latent target proteins within the host system providing an advantageous window for vaccination strategy. With target proteins already within the host, the only ammunition needed is a vaccine boost that redirects the cellular response to target EBV latent proteins. This leads to the production of robust EBV-specific CD8^+^ and CD4^+^ T-cell responses which eventually kills the tumors expressing these proteins (90).

In the last decade, a number of clinical trials on the therapeutic efficacy of vaccination in EBV-associated NPC have shown promising results. The numbers of EBV-associated NPC trials—on clinicaltrials.gov—are approximately 64. This indicates the global interest to unravel the complex interplay of EBV and NPC to merge immunotherapeutic strategies into mainstream clinical practice. A preclinical study conducted by Taylor et al. showed that *in vitro* exposure of dendritic cells to fusion protein containing a carboxyl terminus of EBNA1 with LMP2 in a poxvirus vector led to successful reactivation of LMP2-specific CD8^+^ T cells and EBNA 1-specific memory T cells in healthy seropositive individuals (97). These data initiated two major phase I clinical trials on NPC patients utilizing similar EBV-specific therapeutic fusion vaccine MVA-EBNA1/LMP2 (92, 98). The respective vaccine was produced keeping the immunogenic properties of EBNA1 and LMP2. The vaccine was a functionally inactive fusion protein containing both CD4^+^ and CD8^+^ epitopes (92, 98). Clinical trials utilizing this vaccine were performed in 18 NPC patients (in remission) in Hong Kong with a follow-up study conducted in the UK. Remarkable results were observed with this fusion vaccine in Hong Kong, where threefold to fourfold increase in the magnitude of T cell responses (CD4^+^/CD8^+^) to at least one viral protein in 15 of 18 patients was observed. In some cases, boosting response to both CD4^+^- and CD8^+^-mediated immunity against EBNA1 and/or LMP2 were also observed (98). The vaccine demonstrated a safe immunological profile with low off target toxicities (98). This significantly exceptional result led to a larger follow up study in the UK, in which a total of 14 NPC patients (in remission) were recruited and tested with the same MVA-EBNA1/LMP2 vaccine. Out of 14 patients tested, 8 patients demonstrated an increased CD4^+^ and CD8^+^ responses indicating the reproducible effectiveness and efficacy of this fusion vaccine (92). Due to robust phase I trials data, this vaccine is now being evaluated in a phase II trials involving patients who experience optimal responses to palliative chemotherapy (NCT01094405).

Another type of vaccine development involved the approach of incubating autologous dendritic cells with EBV peptides/viral vectors that express LMP2. In this respect, a clinical study by Lin et al. utilized a cocktail of EBV-specific LMP2 peptides incubated with autologous dendritic cells (99). This vaccine was injected in nine NPC patients of whom, two exhibited enhanced CD8^+^ cellular responses after four injections. Clinically, the cellular responses in the two respective patients also correlated with tumor regression (99). Similar approach was taken by Chia et al. in a phase II trial in which 16 metastatic NPC patients were vaccinated with autologous dendritic cells bearing a truncated LMP1 and a full length LMP2 in an adenovirus vector (100). The vaccine was known as adenovirus-Delta LMP1–LMP2 vaccine and was found to show no increase in CD8^+^ T cell responses, although clinically partial and stable disease was observed in three of the vaccinated patients. The remaining patients showed a delayed type hypersensitivity that did not correlate with any clinical benefit (100). Although robust cellular responses were not observed, the study was the first of its kind to demonstrate the safe profile/tolerance level of EBV vaccines against NPC in humans (100).

Interestingly, vaccine-dependent responses in EBV-associated NPC are cellular only. As such, antigen-specific antibodies for protection against EBV-associated NPC are generally not produced. Therefore, vaccine production against EBV-associated NPC can only be therapeutic and not prophylactic (90).

Results from the EBV-associated NPC vaccine trials have demonstrated many advantages of these therapeutic vaccines (92, 98–100). First, tested vaccines were shown to increase CD8^+^ and CD4^+^ T cell responses in both Chinese and European patients indicating that the vaccine precludes any association with human leukocyte antigen (HLA) variation or EBV strain difference (90, 92). This is important as it paves a wide spectrum of its use in patients with various ethnic/genetic backgrounds. Second, safety studies concluded that these vaccines are well tolerated and produce limited off target toxicities (92, 98–100). Third, these vaccines can be mass produced with highly consistent and reproducible results at a low cost. Finally, minimum trained staff and facilities are required to merge them into clinical practice (91). Though their advantages are well perceived, there are still some limitations associated with these vaccines. The main challenge is to test the vaccines for safety concerns in a larger scale study for a long duration, especially in young patients. This is because EBV-based vaccine requires administration of attenuated full or partial pathogen into the host. In young patients, it is likely that the adverse events may be observed at a later stage of life. Therefore, safety issues, especially in young patients, are a concern that needs to be addressed (91). Furthermore, *in vivo* experimental data generated from testing animal and xenograft models may not be sufficient to be extrapolated for human studies (91).

### Immunotherapy and Virus-Specific T Cells (VSTs) Expansion Methods

Adoptive immunotherapy based on *ex vivo* expansion of antigen-specific T cells has emerged as a powerful and an innovative approach to treat human cancers and viral infections (101, 102). Over the past decade, the manufacturing process for VSTs has been extensively studied aiming to improve the quality of effector cells and increase the speed and the quantity of the production (102). To this end, numerous *in vitro* strategies have been conducted by various groups to identify the best methodology for the expansion of VSTs for prophylaxis or therapy of virus-associated malignancies (103–112).

**Figure 2:**
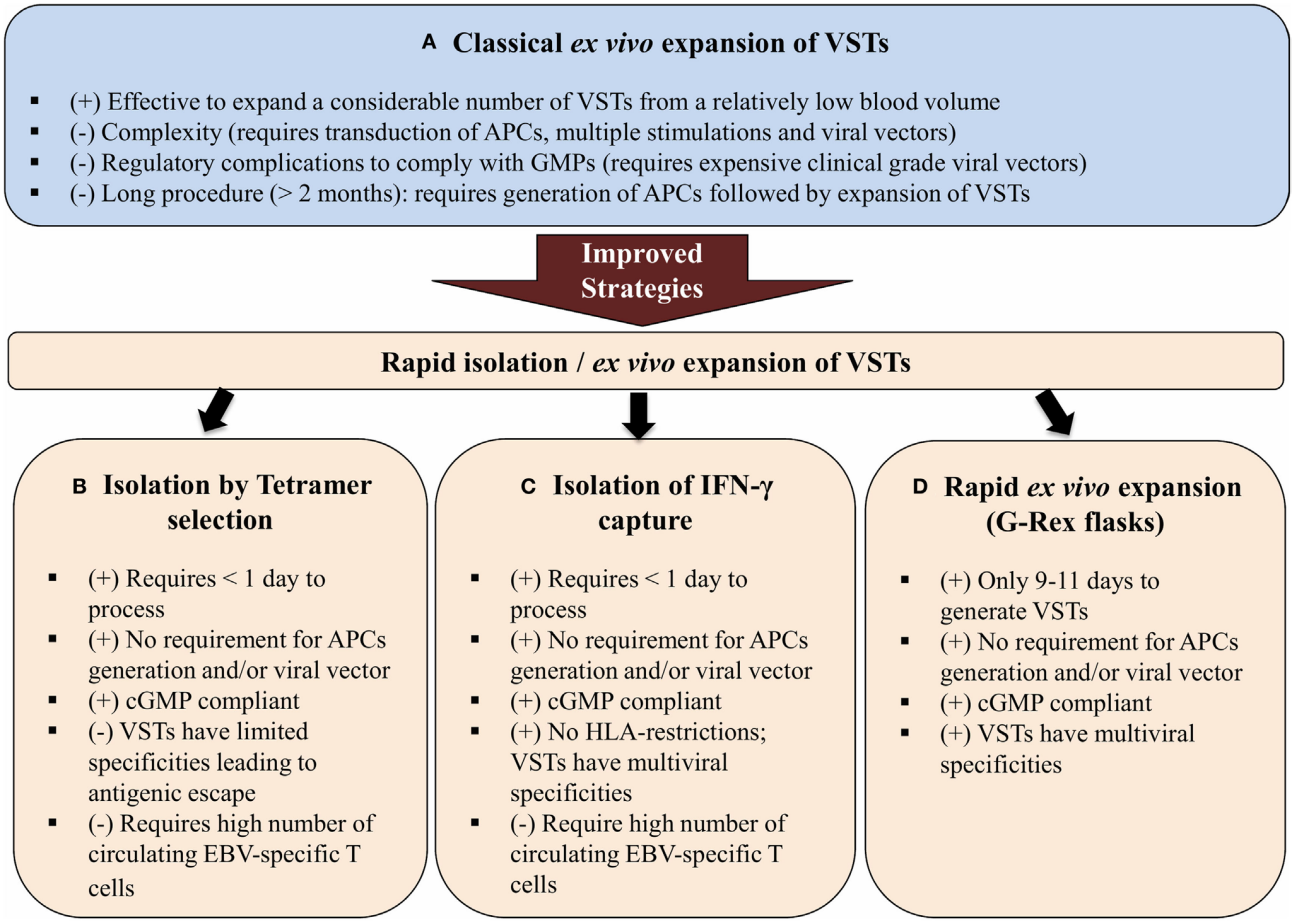
Schematic diagram showing three different improved strategies for the isolation and expansion of VSTs **(B–D)** over the classical *ex vivo* expansion of VSTs **(A)**. Abbreviations: VSTs, virus-specific T cells; APCs, antigen-presenting cells; cGMP, current good manufacturing practices; HLA, human leukocyte antigen; (+) and (−) are, respectively, for advantages and disadvantages of each strategy.

The first experiments for expansion of antiviral T cells for adoptive immunotherapy used antigen-presenting cells (APCs) that had been transduced with either a viral vector or plasmids encoding the antigen of interest. T cells were expanded *in vitro* upon simulation with these APCs. Although effective to expand a considerable number of VSTs, this protocol was difficult to export to clinical use because of the regulatory complications related to complying with current good manufacturing practices (cGMP) (113) (**Figure 2**). Therefore, cGMP-compliant strategies were developed based on the selection of VSTs from bulk donor’s T lymphocytes by a tetramer selection (HLA-restricted tetramer). In this case, T cells are incubated with a tetramer that mimic the viral peptide then are isolated using magnetic beads or fluorescence-activated cell sorting (114–117) (**Figure 2**). This method is rapid, easy, and does not require APCs or exogenous cytokines. However, the tetramer-mediated selection only selects T cells specific for a single HLA-restricted epitope of a single virus and this would allow antigenic escape (118, 119). Another strategy that is able to rapidly generate VSTs is IFN-γ capture. This approach uses an immuno-magnetic separation device to isolate T cells that produce IFN-γ after stimulation by viral antigens. Once the T cells are stimulated, antibodies bind IFN-γ allowing T cells to be isolated by magnetic selection (113). IFN-γ capture is not HLA-restricted and produces a polyclonal product containing both subsets of immune T cells (CD4^+^ and CD8^+^). However, IFN-γ capture and tetramer selection strategies both require seropositive donors and a considerable number of circulating VSTs for clinical use (120) (**Figure 2**).

Various protocols have been developed to manufacture EBV-specific T cell products. These protocols include multimer/tetramer selection, IFN-γ capture, and several methods for *ex vivo* T cells expansion. To date, *ex vivo* expansion is the most commonly used method (120). Initially, *ex vivo* expansion methods used EBV-transformed lymphoblastoid cell lines (LCLs) as APCs. LCLs are important APCs since they express all EBV latency antigens (type III latency) and high levels of class I and II HLA and co-stimulatory molecules (121). Moreover, different groups have developed methods for modifying LCL by either pulsing with synthetic peptide pools encompassing viral antigens or transfecting LCLs with adenovirus vectors that express less immunogenic viral antigens such as LMP1 and LMP2. This strategy helped to increase T cells specificity and promote their cytotoxicity and efficacy in EBV-positive tumors that only express LMP1 and LMP2 (120). Although the activation and expansion of EBV-specific T cells using LCLs is safe and efficacious, the manufacturing process is long. It requires 4–6 weeks to establish LCLs, and then at least 4 weeks to expand EBV-specific T cells followed by 2 weeks for quality control testing to generate a suitable product for clinical use.

Therefore, rapid *ex vivo* culture methods were developed to reduce the manufacturing time to 10–14 days by using a single stimulation by APC pulsed with synthetic peptide pools, or a direct stimulation of PBMCs with synthetic peptide pools. Rapid *ex vivo* culture methods have been used for multivirus-specific T cells, but not for production of cytotoxic T cell products specific only for EBV.

### Rapid T Cell Expansion Strategies

**Figure 3:**
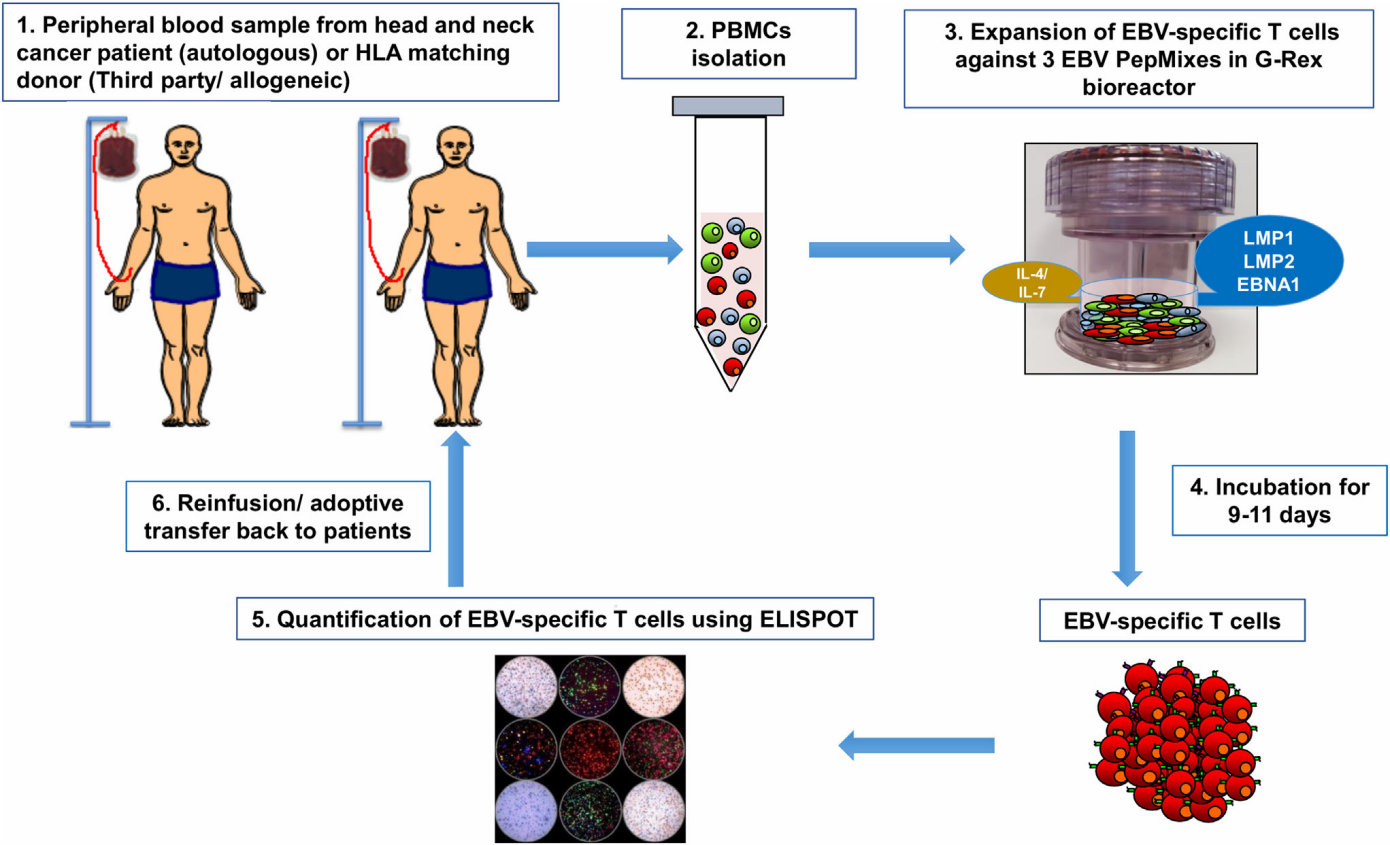
From bedside to bench and back again: Epstein–Barr virus (EBV)-specific T cells can be isolated from peripheral blood of patient with head and neck cancer then reactivated and stimulated *in vitro* to increase their number and promote their specificity (1). If the patient’s antiviral memory T cells are inexistent or their activity is dampened by the immunosuppressive tumor microenvironment (regulatory T cells, myeloid-derived suppressor cells, and/or inhibitory cytokines/chemokines), virus-specific T cells (VSTs) can be obtained from peripheral blood of human leukocyte antigen (HLA)-matching sibling or from third party partially HLA-matched seropositive donor (1). Peripheral blood mononuclear cells (PBMCs) will be isolated from patient/donor peripheral blood by Ficoll-Hypaque density gradient centrifugation (2). To generate EBV-specific T cell lines, PBMCs will be pulsed *in vitro* with a mixture of three overlapping PepMix peptides representing the EBV viral antigens (latent membrane protein 1, latent membrane protein 2, and EBV-induced nuclear antigen 1) present on nasopharyngeal cancer (NPC) tumor cells. PBMCs are suspended in specific culture media supplemented with IL4 and IL7 and then transferred to a G-Rex^®^ culture device (3). After 9–11 days of culture, VSTs are harvested and assessed for viability and quantity (4). The viral specificity of these T cells will be assessed by ELISPOT assay (5). The expanded EBV-specific T cells obtained from the patient or the HLA-matching donor will be infused back onto patient peripheral blood (6) as autologous and allogeneic adoptive T cell therapy, respectively.

To avoid the need for viral vectors, Gerdemann et al. developed a rapid expansion strategy in which small numbers of donor PBMCs were stimulated for 10 days with autologous dendritic cells DC previously transfected with DNA plasmids that express EBNA1, LMP2, and BZLF1 in the presence of IL-4 and IL-7. The total procedure required 17 days, including 7 days for DC generation (122, 123). This rapid expansion strategy was shortened by using overlapping peptide libraries (pepmixes) that represent the viral antigen(s) of interest instead of plasmids (113, 124). These pepmixes are pulsed directly onto PBMCs eliminating the requirement for DCs. APCs present in donor’s PBMCs stimulate the T cells to grow. When coupled with a G-Rex^®^ gas-permeable culture device, VSTs are obtained in 9–11 days and are ready for infusion into patient peripheral blood after quantification and quality control testing (Figure 3). This novel gas-permeable culture device G-Rex^®^ (Wilson-Wolf Manufacturing, Minneapolis) has been designed to support optimal cell growth through improved gas exchange. It has recently been used for GMP-compliant functional T cell expansion in different studies (104, 125–128). Recently, experimental studies carried out by Leen et al. implemented a new rapid protocol and reported data on the development and clinical activity of single preparations of multivirus-specific T cells. The preparations were made by direct stimulation of PBMCs with overlapping peptide libraries that incorporated five viral antigens including EBV coupled with culture in G-Rex^®^ devices for optimal T cell expansion (125). The expanded VSTs met the desired specifications of multiviral specificity, rapid production, and sustained broad antiviral activity (125). This rapid protocol uses G-Rex^®^ culture permeable system that effectively supports the expansion of VSTs and increases output by 20-fold while decreasing the required labor time (129). In addition, specific interleukins (IL-7 and IL-4) were incorporated to, respectively, inhibit apoptosis and promote expansion of these VSTs in 10 days (130). Moreover, the pepmixes tool to generate VSTs represents robust technology. Gerdemann and colleagues have also expanded *ex vivo* multivirus-specific T cells recognizing seven viruses indicating that there is no obvious limit to the number of virus antigens that could be incorporated in this technology (104, 131).

Despite the advances in the manufacturing process for the generation of VSTs, none of the approaches described above are able to expand such T cells from virus-seronegative donors. Indeed, several groups have developed strategies to stimulate naïve T cells present in cord blood (132, 133). In this respect, cord blood-derived T cells were expanded to sufficient numbers for clinical application using the G-Rex^®^ gas-permeable cell culture flask. It was demonstrated that it is possible to generate multivirus-specific T cells in a virus-inexperienced setting compliant to cGMP (129, 134).

Other approaches are being developed to improve the antitumor activity of EBV-specific T cells including genetic approaches to enhance the resistance of these cells toward the immunosuppressive tumor microenvironment, in addition to combination approaches with other immune-modulating modalities (immune checkpoints such as CTLA-4 blockade or PD-1/PD-L1 blockade) (135). Indeed, clinical observations have suggested that PD-L1 antigen is expressed on NPC tumor cells and may be associated with a poor outcome in NPC. Moreover, an upregulation of PD-1 antigen was observed on expanded EBV-specific T cells. These observations suggest that PD-1/PDL-1 blockade could enhance the activity of EBV-specific T cells in treating NPC patients (10, 53, 136).

### Adoptive VST Therapy in EBV-Related Head and Neck Cancers

Adoptive transfer of EBV-specific cytotoxic T cells has been suggested as an adjunct to conventional treatment in attempt to provide an effective prophylaxis and treatment of EBV-positive malignancies. EBV-positive NPC cells express subdominant EBV antigens (EBNA1, LMP1/2) providing potential target antigens for EBV-specific cytotoxic T cells. Interestingly, T cells specific for LMP2 and LMP1 antigens were found in the peripheral blood of NPC patients and could therefore potentially be isolated, stimulated, and expanded for immunotherapeutic approaches (137–141). In fact, many recent studies have shown that adoptive T cell therapy using *ex vivo* generated EBV-specific cytotoxic T cells could be effective in the prophylaxis and the treatment of EBV-associated head and neck malignancies such as NPC (9, 142, 143).

The first reported use of EBV-specific cytotoxic T cells was presented in 1998 by Roskrow et al. who had expanded cytotoxic T cells from patients with Hodgkin lymphoma. The results showed that the infusion of these cells into patients resulted in a clinical antiviral activity *in vivo* and in a lower EBV DNA loads in these patients’ blood (144). More recently, Bollard et al. had expanded autologous T cells specific to the LMP1 and LMP2 from patients with EBV-associated lymphoma. They showed that these expanded EBV-specific T cells could induce durable complete responses in these patients with minimal side effects (145). The first reported study using EBV-specific T cells in treating head and neck carcinomas was carried out by Chua et al. In this study, four patients with advanced NPC received autologous EBV-specific T cells. A decrease in EBV viral load in the plasma was observed in three patients without any adverse effect (140). Later, a phase I clinical study showed that treatment of patients with relapsed NPC with autologous EBV-specific T cells induced antitumor clinical responses in 6 out of 10 patients (146). At the same time, the results of a study of 10 patients diagnosed with advanced NPC demonstrated that adoptive transfer of autologous EBV-specific CTLs is safe and can be associated with significant antitumor activity (137). Similarly, a study of 24 patients with metastatic forms of EBV-positive NPC showed that EBV-specific T cells were successfully expanded from 16 patients (72.7%). Besides, the adoptive transfer of these EBV-specific T cells resulted in long-term clinical benefits with no significant toxicity (142). Another phase I/II clinical trial assessed the effect of EBV-specific T cells in refractory NPC and showed antitumor activity in patients with locoregional NPC, while a limited clinical response was observed with metastatic NPC (147). Recently, a phase II clinical study involved 35 patients with advanced recurrent or metastatic NPC who received first-line treatment with chemotherapy followed by adoptive transfer of EBV-specific T cells. This resulted in a response rate of 71%, with increased survival rates up to 63% (143). Very recently, Smith et al. studied the use of an adoptive cellular therapy targeting the LMP1/2 and EBNA1 antigens expressed in NPC. They generated LMP/EBNA1-specific T cells using the adenovirus AdE1-LMP poly vector which promoted optimal expansion of viral-specific T cells from low frequency precursors. They observed that autologous LMP/EBNA1-specific T cells could be generated from the majority of patients with EBV-positive NPC. Their results showed that NPC stabilization was associated with the number of LMP/EBNA1-specific T cells administered to the patient. This group also suggested the importance of an allogeneic “off-the-shelf” production of LMP/EBNA1-specific T cells in an attempt to increase the frequency and efficacy of these cells to enable their clinical use in the treatment of NPC (10). All these observations indicate that adoptive transfer of EBV-specific T cells has a promising clinical outcome in patients with EBV-positive NPC and should be suggested as a complementary therapy following conventional NPC treatments especially in recurrent and metastatic forms of the disease where the patients are less responsive to chemotherapy.

## Conclusion and Future Prospects

Evidently, EBV plays a complex and an intricate role in the pathogenesis of NPC. The viral proteins, particularly LMP1, LMP2, and EBNA1 are involved in the modulation of the key factors contributing to malignant transformation. They are capable of exerting control at every stage of the cancer from initial oncogenesis and tumor initiation to tumor progression and metastasis. These proteins participate in the regulation of important signaling pathways through modulating the activity of kinases. In addition, they can interact with acclaimed critical cancer-related proteins. Apart from employing mechanisms to initiate oncogenesis by the transformation of normal cells to tumors, they can further sustain the cancer by displaying complex mechanisms of immune escape. They achieve this by interacting with and by modulating certain immune-checkpoint inhibitors. In addition, miRNAs are found to be encoded by the EBV genome and to contribute further to regulating oncogenic activity at the post-transcriptional level. However, despite the varying mechanisms employed by the EBV proteins in propagating NPC cancer, the advancements in the development of novel immunotherapies is seemingly promising to evade the oncogenic properties of the virus. Although therapeutic vaccines against EBV-associated NPC seem ideal, there is always a need to explore combination with other therapies, a mainstay of classical successful treatment strategies. Future prospective trials focusing on the role of radiotherapy/chemotherapy in combination with therapeutic vaccines may potentiate robust antitumor responses to control tumor. Furthermore, novel therapeutics including immune-checkpoint inhibitors, such as anti-PD-1/anti-PD-L1, in combination with therapeutic vaccines may unleash the immune response against EBV-associated NPC leading to improved survival and tumor management. It is also worth directing therapeutic research toward novel EBV proteins that may be able to generate EBV-associated neutralizing antibodies. In addition, although the application of T cells immunotherapy targeting EBV antigens was shown to be successful in patients with NPC, this approach provides a challenge as only subdominant EBV antigens are expressed by these malignancies. Current protocols for preparation of EBV-specific T cells should be improved to overcome the generation of tumor escape mutants, down regulation of MHC class I expression on tumor cells, and the presence of inhibitory T cells at the tumor site. To this end, additional specificities could be engrafted onto EBV-specific T cells through the expression of chimeric antigen receptor which would bind to specific tumor antigens expressed by the tumor cells. CD70 was previously suggested as a candidate antigen for NPC (148). Additional approaches are being developed to improve the antitumor activity of EBV-specific T cells; genetic approaches (149) were applied to enhance the T cells resistance to immunosuppressive factors of the tumor microenvironment, such as inhibitory cytokines and chemokines secreted by malignant cells which downregulate T cells proliferation and function. Another used approach is the combination with immune-checkpoint blockade (CTLA-4 blockade or PD-1/PD-L1 blockade). Finally, T cells specific to LMP1 and 2 are observed in peripheral blood of NPC patients. However, a focus on a production of third party banks by expanding specific T cells from HLA-matching donors would have an important impact on treating NPC patients who present weak or inexistent EBV-specific T cells (Figure 3).

## Author Contributions

QF, MM, AR, VI, NA, AG, SU, and SD were involved in writing specific parts of the manuscript. QF was involved in writing the sections on the EBV infection, viral oncogenic pathogenesis, and EVB protein expression. MM and AR contributed to the immunotherapy section on adoptive T-cell transfer and EBV vaccines, respectively. VI contributed toward designing the required illustrations and in tabulating information for concise presentation in this manuscript. NA, AG, and SU contributed toward the general critical writing and editing of various sections of this paper. SD was involved in conceiving, defining, directing the framework of the manuscript and provided overall supervision in bringing this manuscript together. All the authors read and approved the manuscript for publication.

## Conflict of Interest Statement

The authors declare that the research was conducted in the absence of any commercial or financial relationships that could be construed as a potential conflict of interest.
